# mtDNA sequence variants in subtypes of epithelial ovarian cancer stages in relation to ethnic and age difference

**DOI:** 10.1186/1746-1596-3-32

**Published:** 2008-07-28

**Authors:** Felix O Aikhionbare, Sharifeh Mehrabi, Winston Thompson, Xuebiao Yao, William Grizzle, Edward Partridge

**Affiliations:** 1 Department of Medicine, Morehouse School of Medicine, Atlanta, GA, 30310, USA; 2 Department of Obstetrics and Gynecology, Morehouse School of Medicine, Atlanta, GA, 30310, USA; 3 Department of Physiology, Morehouse School of Medicine, Atlanta, GA, 30310, USA; 4 Department of Pathology, University of Alabama, Birmingham, AL, 35294, USA; 5 Comprehensive Cancer Center, University of Alabama Birmingham, AL, 35294, USA

## Abstract

Epithelial ovarian cancer is the fifth leading cause of cancer mortality among women in the United States. For this disease, differences in age-adjusted incidence and survival rates between African American and Caucasian women are substantial. The objective of this study was to examine mtDNA sequence variants in 118 frozen tissues of three subtypes of epithelial ovarian cancer (serous, n = 48 endometrioid, n = 47 and mucinous, n = 23) and matched paracancerous normal tissues (n = 18) in relation to racial/ethnic and age differences. Restriction fragment length polymorphism (RFLP) and polymerase chain reaction (PCR)-based sequencing were used to evaluate two regions of mtDNA spanning 5317 to 7608 and 8282 to 10110 bp and including *ND *subunits 2, 3, *MT-COI, II*, and *III, ATPase *8, a part of *ATPase *6, and *tRNA *genes in frozen ovarian tissues obtained from the southern regional Cooperative Human Tissue Network (CHTN) and University of Alabama-Birmingham (UAB) Ovarian Spore Center. Thirty-nine mtDNA variants were detected of which 28 were previously unreported. One somatic variant of C9500T was observed. A variant, C7028T in the *MT-CO1 *gene, had an ascending frequency from borderline (8%) to stages III/IV (75%) among the three ovarian cancer subtypes and stages. It was found in 86% (42/49) of African-American and 43% (37/87) of the Caucasian women. A variant, T8548G in the *ATPase 6 *gene was detected at a frequency of 72% (18/25) in ovarian serous subtype tissues in stages III/IV. Of the African American patients under age 40, 95% (20/21) harbored the T8548G variant; this was in contrast to only 22% (8/35) of Caucasian patients in same age group. Variants C7256T and G7520A had a frequency of 54% (6/11) in endometrioid stage III; no corresponding variants were observed in mucinous subtype stage III. Furthermore, variants C7256T and G7520A were absent in serous ovarian cancer subtype. Interestingly, the C7520T variant in *tRNA *gene was present in 74% (36/49) of African American and 26% (23/87) of Caucasian patients. Taken together, our results suggest that, with respect to ethnic and age difference, these mtDNA variants may be involved in epithelial ovarian carcinogenesis.

## Findings

Although early diagnosis provides the best chance for cure in patients with epithelial ovarian cancer, the lack of appropriate diagnostic strategies continues to confound clinical oncologists. Hence, there is a pressing need for a specific, less-invasive procedure to allow accurate subtyping and staging evaluation of epithelial ovarian cancers for early detection and treatment strategies. Most ovarian cancers occur in older women, and mtDNA mutations are thought to have a causal role in many age-related pathology because of mitochondria involvement in cell apoptosis and reactive oxygen species (ROS) activities [1-5]. Mitochondrial gene products are essential for normal cell functions and all mitochondria within a cell share identical DNA sequence. MtDNA sequence within the same cell may be preferentially modified by carcinogens and mtDNA is repaired less efficiently compared with that of nuclear DNA [6], as a result, it has been implicated in carcinogenesis [2-5,7].

Sequence variants in certain regions of mtDNA have been associated with ovarian cancer [7,8]. Between African-American and Caucasian women with this disease, there are differences in age-adjusted incidence and survival rates [9]. Nevertheless, definitive mtDNA sequence variants in epithelial ovarian carcinogenesis in relation to racial/ethnic and age differences have yet to be established. Given the connection between mitochondria, ROS, and neoplasia, mtDNA from three subtypes of ovarian cancer stages (including the borderline and cystadenoma) and matched paracancerous normal tissues were screened for mtDNA sequence variants that might be used as potential prognostic markers for ovarian carcinogenesis in relation to racial/ethnic and age differences. We speculated that there is an association between one or more mtDNA sequence variants and ovarian cancer incidence with respect to differences in ethnicity and age. In this study, we analyzed sequence variants in two regions of mtDNA obtained from 118 epithelial ovarian cancer and 18 matched paracancerous normal tissues spanning 5317 to 7608 and 8282 to 10110 bp, including *ND *subunits 2 and 3; *MT-COI*, *II*, and *III*; *ATPase *8; part of *ATPase *6; and *tRNA *genes.

All studies were implemented under protocols approved by Institutional Review Boards of Morehouse School of Medicine and UAB.

One hundred and eighteen frozen epithelial ovarian cancer tissues from three histologic subtypes [(serous n = 48; endometrioid, n = 47; mucinous n = 23), including stages I-IV, benign cystadenomas, borderline tumors] and 18 matched paracancerous normal tissues that paired with some of the tumors were obtained from CHTN and UAB-Ovarian Spore Center. Ovarian cancer subtype and clinicopathologic stage tissues were histologically determined based on the criteria outlined by AJCC. Of the tissues, 49 of the tissues were African American and 87 tissues were Caucasian women. The mean age of the patients was 51.3 ± 5.7 years. MtDNA was isolated from the frozen tissues using centrifugation according to the manufacturer's protocols (BioVision, Research Products). Total mtDNA was quantified and diluted to 50 ng/μl for PCR reaction. MtDNA variants were detected by use of RFLP and PCR-based sequence as previously described [7]. Sequences of both sense and anti-sense strands were derived with an ABI 3100 Genetic Analyzer. Sequences were aligned and compared to mitochondrial DNA sequences [GenBank: J01415] in relation to ovarian cancer subtypes, stages, ethnicity and age of the patients. The MITODAT database was used to determine sequence variants.

**Figure 1:**
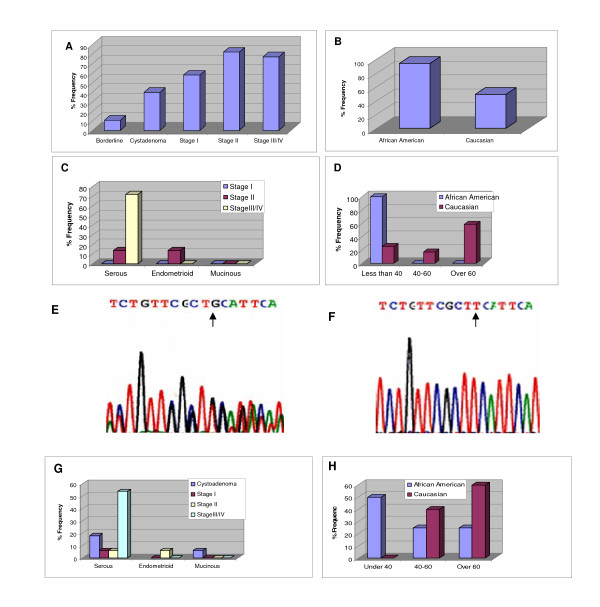
Graph illustrating the differences in ethnicity, age and the number of mtDNA sequence variants obtained from three subtypes (serous, endometrioid and micinous) of epithelial ovarian cancer stages using following primers; *F 5'-CCACCATCACCCTCCTT-3', and R 5'-CCTACTTGCGCTGCATGTGCC-3' and F 5'-CCCCTCTAGAGCCCACTGTAAAGC-3', R 5'-GTAGTAAGGCTAGGAGGGTG-5*; (A), Cumulative frequency of the C7028T variant observed in three subtypes of epithelial ovarian cancer stages; B, ethnic related frequency of C7028T variant observed between African American and Caucasian women with ovarian cancer; C, frequency of T8548G variant among the three subtypes and stages of epithelial ovarian cancer; D, frequency of T8548G variant observed in relation to age between African American and Caucasian women with ovarian cancer; E, electropherogram indicating nucleotide G observed in some of the three epithelial ovarian cancer tissues; F, electropherogram indicating nucleotide T in all the colorectal cancer tissues tested as positive controls; G, frequency of the C9500T variant in relation to stages of the three epithelial ovarian cancer ; H, frequency of the C9500T variant in relation to age between African American and Caucasian women with ovarian cancer.

Sequence analyses revealed the presence of thirty-nine variants, of which 28 were previously unreported [see Additional file 1]. These previously reported variants were observed: C7028T, C7256T, G7520A, T8548G, T8588C, A8860G, C9488G, C9500T, T9540C, C9857T, T9951C, 10045delA. Some of the unreported variants were G7520A, T8548G, C9488G, C9500T, C9857 and T9951C; frequencies ranged from 41–93 percent. Additionally, a somatic variant of C9500T was observed. Variants C7028T 82/136 (60%) and A8860G 125/136 (93%) were evenly distributed in the three ovarian cancer subtypes and stages. The variant C7028T in the *MT-CO1 *gene, which had an ascending frequency from borderline (8%) to stages III/IV (75%) among the three ovarian cancer subtypes and stages, was found in 86% (42/49) of African-American and 43% (37/87) of Caucasian patients (figures 1A and 1B). Although germ-line mutations in the *MT-COI *gene of mtDNA are thought to influence the incidence of prostate cancer in African American men [4], it is unclear whether this particular variant of C7028T is involved in epithelial ovarian carcinogenesis as one of the genetic risk factors for racial/ethnic differences in ovarian disease. The present evidence, in spite of the limited sample size, is consistent with such involvement. A variant, G8860A, was observed in the *ATPase 6 *gene at a frequency of 93%. Even though this *ATPase 6 *gene influenced the ROS productions [2,4-6], it is not known if this mutation contributes to a common mechanism related to the onset and progression of epithelial ovarian cancer subtypes and stages.

The most striking aspect of our results regarding age groups is that the T8548G variant in the *ATPase 6 *gene was present at a frequency of 72% (18/25) among the subtype of serous ovarian cancer stages III/IV. Of African-American patients under the age 40 with serous ovarian cancer, 95% (20/21) harbored the T8548G variant compared to 22% (8/35) of Caucasian patients of same age group (figures 1C and 1D). Notably, variant T8548G was not detected in the 14 samples of colorectal cancer tested as "positive controls" (figures 1E and 1F).

Moreover, the somatic variant C9500T occurred with a frequency of 52% (12/25) in serous ovarian cancer stages III/IV. Of African American patients under age 40, 47% (10/21) harbored the variant, compared to 1% (2/35) of Caucasian patients. Among Caucasian patients over age 60, however, the frequency of the C9500T variant was 60% (21/35), compared with 23% (5/21) for African American patients (figures 1G and 1H). Although the function of these mtDNA variants (perhaps in combination with other mtDNA mutations) in relation to the age-specific racial/ethnic difference is unclear, ovarian cancer generally occurs after menopause and is related to old age [10]. Nevertheless, these findings support the concept that there is a pronounced ethnic difference for younger and older women with more advanced ovarian cancer [9]. Furthermore, African American patients under age 40 with this disease have more rapid progression and poorer survival rates compared with Caucasian patients [11]. Random genetic drift may explain the accumulation of mtDNA mutations with age [12]. Age is an important risk factor for ovarian cancer: approximately 70% of cases occur in women over the age of 55 [13]. In the present study, the patients' age at the time of surgery ranged from 28 to 71 years (median age 51.3). These variants may function in combination with other risk factors [9] in the advancement of serous ovarian cancer in African American patients relative to Caucasian patients.

Comparison of sequence variants among the three ovarian cancer subtypes revealed a combined variant, C7256T and G7520A, with a frequency of 54% (6/11) in endometrioid stage III, but there was no correspondence in incidences in mucinous subtype stage III. In addition, variants C7256T and G7520A were absent in serous ovarian cancer subtype. The C7520T variant in *tRNA *gene occurred in 74% (36/49) of African American and 26% (23/87) of Caucasian among patients. Furthermore, a sequence variant T9540C in the *MT-COIII *gene was observed at a high frequency in 88% (42/49) of African American patients compared with Caucasian patients at 10% (9/87); this variant was not found in the mucinous subtype samples. We had expected that, in many cases, the mtDNA sequence data in the three ovarian cancer subtypes would be similar. Because the three epithelial tumors (serous, mucinous, and endometrioid) are thought to share a common cellular ancestry with other structures of the reproductive tract and since metaplasia of the cyst lining give rise to growths of serous, mucinous, or endometrioid cells [14]. Moreover, mtDNA mutations in the *tRNA *gene are highly conserved [6] and accumulate with age, particularly during the increased mitotic and post-mitotic activities accompanying ovulation and repair [15]. Nevertheless, it possible that etiologic features of the common epithelial ovarian cancer subtypes differ because of development from cells that already histological differentiated [16].

In this study, we have observed that, in regard to mtDNA from African American women and Caucasian women with epithelial ovarian cancer, there is an appreciable difference in the frequency of sequence variants in relation to age. As a result of the fact that mtDNA sequence variants may accumulate as ovarian cancer progresses through difference stages, mtDNA and mitochondria may be involved in the process of epithelial ovarian carcinogenesis. Based on this study and others, we suggest that the epithelial ovarian cancer subtypes (serous, mucinous and endometrioid) may be etiologically unrelated and thus should be considered as difference entities. However, larger, population-based studies are required to quantify the functional role of mtDNA sequence variants in histological subtypes and stages of ovarian cancer. Since we sequenced only a 4.1 kb fragment of the 16.5 kb mitochondrial genome, the number of mtDNA sequence variants that could correlate with ovarian tumor subtypes and stages may exceed the number we observed.

## List of abbreviations

 AJCC: American Joint Committee on Cancer; ATPase: ATP synthase 8; COX: Cytochrome c Oxidase; ND: NADH dehydrogenase; mtDNA: Mitochondrial DNA; MITOMAP: Mitochondria databank; OXPHOS: Oxidative phosphorylation; PCR: Polymerase Chain Reaction; ROS: Reactive Oxygen Species; CHTN: Southern Regional Cooperative Human Tissue Network; UAB: University of Alabama-Birmingham.

## Competing interests

The authors declare that they have no competing interests.

## Authors' contributions

FOA conceived, designed and coordinated the study and participated in data analysis and drafted the manuscript. MS participated in acquisition of data and drafting the manuscript. WT helped to draft the manuscript and participated in its review. XO participated in the review of the manuscript. WG: provided the clinical samples and participated in the review of the manuscript. ED provided the initial clinical samples and participated in the review of the manuscript. All authors read and approved the final version.

## Supplementary Material

Additional file 1 Mitochondrial DNA variants obtained from the three epithelial ovarian cancers (serous, endometrioid and mucinous). The data provided represent the mitochondrial sequence variants spanning 5317 to 7608 and 8282 to 10110 bp, including *ND *subunits 2, 3, *MT-COI, II, III*, *ATPase 8*, a part of *ATPase *6, and *tRNA *genes. Click here for file
