# Supplementary material for: mtDNA sequence variants in subtypes of epithelial ovarian cancer stages in relation to ethnic and age difference
Source: Diagn Pathol. 2008 Jul 28;3:32. doi: 10.1186/1746-1596-3-32 (PMC2494992; doi:10.1186/1746-1596-3-32)
Supplement: Additional file 1 — Mitochondrial DNA variants obtained from the three epithelial ovarian cancers (serous, endometrioid and mucinous). The data provided represent the mitochondrial sequence variants spanning 5317 to 7608 and 8282 to 10110 bp, including ND subunits 2, 3, MT-COI, II, III, ATPase 8, a part of ATPase 6, and tRNA genes. [file 1746-1596-3-32-S1.doc]

Additional file 1. mtDNA variants in three epithelial ovarian subtypes

| Mt region/genes | Nucleotide position |
| --- | --- |
| MT-ND2* | G5460A |
| MT-ND2* | G5471A |
| MT-TA* | C5603T |
| MT-TA** | A5612C |
| MT-TA* | T5655C |
| MT- NC5* | CCins at 5899 |
| MT-CO1* | G5949A |
| MT-CO1** | G5950A |
| MT-CO1** | C5953A |
| MT-CO1** | A6022G |
| MT-CO1** | Tins at 6044 |
| MT-CO1** | C6054T |
| MT-CO1* | C7028T |
| MT-CO1** | C7029G |
| MT-CO1** | C7174T |
| MT-CO1* | C7256T |
| MT-CO1** | C7275T |
| MT-TD** | C ins at 7505 |
| MT-TD** | G7520A |
| MT-TD** | A7523G |
| MT-ATP8** | A8399G |
| MT-ATP8** | A8411G |
| MT-ATP** | T8548G |
| MT-ATP6* | T8588C |
| MT-ATP6* | A8860G |
| MT-CO3 ** | C9391A |
| MT-CO3 ** | A9436T |
| MT-CO3 ** | G9438A |
| MT-CO3 ** | C9441A |
| MT-CO3 ** | C9485G |
| MT-CO3 ** | C9488G |
| MT-CO3 ** | T9496C |
| MT-CO3 ** | C9500T |
| MT-CO3 * | T9540C |
| MT-CO3 ** | T9601C |
| MT-CO3 ** | C9520G |
| MT-CO3 ** | A9855G |
| MT-CO3 ** | C9857T |
| MT-TG ** | T del 10045-10046 |

*Previously reported mtDNA variants and ** unreported mtDNA variants

based on Mitomap database.
